# High‐fidelity and iterative affinity extraction of hyaluronan

**DOI:** 10.1002/pgr2.70008

**Published:** 2024-12-06

**Authors:** Dorothea A. Erxleben, Felipe Rivas, Ian Smith, Suruchi Poddar, Paul L. DeAngelis, Elaheh Rahbar, Adam R. Hall

**Affiliations:** ^1^ Virginia Tech‐Wake Forest University School of Biomedical Engineering and Sciences, Wake Forest University School of Medicine Winston‐Salem North Carolina USA; ^2^ Department of Biology Wake Forest University Winston‐Salem North Carolina USA; ^3^ Department of Biochemistry and Physiology University of Oklahoma Health Sciences Center Oklahoma City Oklahoma USA; ^4^ Departments of Biomedical Engineering and Veterinary Physiology and Pharmacology Texas A&M University College Station Texas USA; ^5^ Comprehensive Cancer Center, Wake Forest University School of Medicine Winston‐Salem North Carolina USA

**Keywords:** biomarker, hyaluronan, microfluidics, nanopore, nanosensing

## Abstract

The glycosaminoglycan hyaluronan (HA) serves a variety of crucial physiological functions in vertebrates. Synthesized at the plasma membrane and secreted into the extracellular environment, HA polymers span a wide range of molecular weights (MW) that define their activity through a notable size‐function relationship. Analytical technologies for determining HA MW distributions typically require selective extraction from complex biofluids or tissues. A common method for achieving this is immunoprecipitation‐like pull‐down using specific HA‐binding proteins bound to magnetic beads. Here, we present a systematic investigation of experimental variables involved in this process, leading to an affinity extraction protocol that enables iterative bead reuse and reagent lifetime maximization, thereby enhancing the efficiency of the HA extraction process. Our methods provide a framework for general optimization of immunoprecipitation in other contexts with heterogenous analyte sizes.

**Abbreviations**
ECD: event charge deficit
GAG: glycosaminoglycan
HA: hyaluronic acid
MW: molecular weight
PBS: phosphate buffered saline
PMMA: polymethyl methacrylate
SEC: size exclusion chromatography
SSNP: solid‐state nanopore
VG1: versican G1 domain

## INTRODUCTION

Hyaluronan (hyaluronic acid, or HA) is a nonsulfated glycosaminoglycan (GAG) that is synthesized at the plasma membrane and secreted into the extracellular environment where it plays diverse roles that range from structural tissue support and cell motility^1^ to leukocyte adhesion and intracellular signaling.^2^ The molecular weight (MW) of HA can vary from small fragments below 10 kDa to polymers of significant size (6–8 MDa).^3^, ^4^ Notably, this broad MW range is accompanied by a strong size‐function relationship wherein, in general, low MW or fragmented HA is associated with pro‐inflammatory processes, tissue remodeling, and angiogenesis while high MW HA is associated with protective, anti‐inflammatory, and immunomodulatory behaviors.^5^, ^6^, ^7^ These contrasting functions have positioned both the quantity and the size distribution of HA as potential biomarkers of pathophysiology.^8^, ^9^, ^10^, ^11^

Analytical approaches for HA size assessment include viscometry, size exclusion chromatography (SEC), multi‐angle or dynamic light scattering, and gel electrophoresis. While each of these methods has been employed successfully, they have also revealed limitations that range from narrow resolution to a requirement for large HA sample amounts that can be difficult to obtain, as well as inform only the mean MW values.^12^ As an alternative, we have established the solid‐state nanopore (SSNP) platform for the sensitive, high‐resolution analysis of HA MW distributions from small sample masses.^13^, ^14^ However, a challenge with SSNPs as well as SEC and some other HA size analysis techniques is the need for high‐quality analyte extraction (or purification). Because these methods lack intrinsic analyte detection selectivity, pretreatments must be employed to isolate or extract HA from complex matrices and ensure that other constituents do not interfere with the results. Mechanical, biochemical, and chemical techniques can all be used to extract HA from complex biological samples with specific protocols depending on the properties of the starting material and the desired purity and yield. For example, cells and aggregates can be removed by centrifugation, proteins and nucleic acids can be digested enzymatically, and lipids can be removed via solvent extraction. Isolation of HA from other GAGs can in principle be achieved by digestion of off‐target species, but limited specificities and reaction efficiencies of relevant enzymes challenge this approach. Additionally, HA can be extracted by chemical fractionation or ion exchange chromatography but these methods necessitate multiple processing steps and large sample mass, require expensive equipment, and can still result in contaminants.^12^

Recently, the extraction of HA using silica particle solid phases was demonstrated^15^ where high ionic strength solutions (e.g., 4–6 M NaCl) could promote the binding of HA to silica. Since other polyanions like sulfated GAGs and nucleic acids can also bind with silica at some salt concentrations, targeted pretreatments were needed to limit contaminants. However, variability in the precise conditions required to remove specific contaminants could pose a challenge for increasingly heterogenous biological matrices where diverse molecules can be present at variable concentrations or where additional contaminants must be considered. Moreover, this technique may not provide binary selection of HA in all circumstances and could result in the retention of trace contaminants. An alternative approach for selective HA purification is affinity extraction in which the isolation principles of immunoprecipitation are applied. In the general method, superparamagnetic beads are decorated with highly specific binding agents. When introduced to a complex mixture, these beads bind only to the target biomolecules, enabling their magnetic isolation and subsequent elution. While immunoprecipitation typically employs antibodies for the capture process, specificity for HA was first achieved^16^ by incorporating the G1 domain of the link module superfamily protein versican (VG1),^17^ a high‐affinity hyaladherin with no significant off‐target recognition for other GAGs. This bead‐based affinity extraction of HA using VG1 was applied to human milk^16^ and later adapted to other complex matrices.^13^, ^18^, ^19^, ^20^, ^21^

Despite its effectiveness at retaining pure HA, key challenges remain for the implementation of this bead‐based approach. These include the irreversibility of the conventional elution process, the limited lifespan of the protein reagent, and the dependence of capture on passive HA diffusion. In this work, we present methodological adaptations of the affinity extraction protocol that address each of these challenges. First, we describe non‐destructive elution of HA using high salt conditions, enabling iterative reuse of the VG1‐beads. Next, we employ conditions that extend reagent lifetime without loss of extraction efficacy by minimizing incubation times and exposure to ambient conditions. Finally, we describe a microfluidic device capable of supporting flow‐based delivery of HA to the binding matrix, thereby providing a critical step towards automating the extraction processes and facilitating the use of large‐volume samples. Collectively, these modifications yield a set of high‐fidelity HA extraction methods that incorporate reusable reagents for improved efficiency.

## MATERIALS AND METHODS

### Hyaluronan samples

Lyophilized polydisperse HA (hyaluronic acid sodium salt from *Streptococcus zooepidemicus*, H9390; Sigma‐Aldrich) was suspended as received at a concentration of 1000 ng/µL in ultra‐pure water and used as a stock solution to produce all HA samples through dilution. No further purification was performed. An average SSNP calibration curve^13^ was generated by measuring quasi‐monodisperse samples of HA^22^ (Hyalose, LLC) having mean MWs of 111, 545, and 1076 kDa and varying within 5% of the reported mean (polydispersity = 1.001–1.035, as estimated by size exclusion chromatography with multilaser light scattering) across a range of SSNPs.

### Affinity extraction of HA

HA samples were extracted using biomagnetic precipitation as reported previously.^13^, ^23^ Superparamagnetic beads (Dynabeads™ M‐280 Streptavidin, 11206D; Thermo Fisher Scientific) were washed according to the manufacturer's directions and incubated with biotinylated VG1 (bVG1, G‐HA02; Echelon Biosciences) at a ratio of 1 µg bVG1 per 100 µg of beads in 150 µL of 1× phosphate buffered saline (PBS; 11.9 mM phosphates, 137 mM sodium chloride, 2.7 mM potassium chloride; pH 7.4) for 1 h at room temperature. 50 µL of polydisperse HA at 20 ng/µL in 1× PBS was then added to a 50 µL aliquot of VG1‐beads and incubated at room temperature or refrigerated conditions, as indicated in the text. Resulting HA‐bound beads were isolated with a magnet and washed to remove unbound material. Beads were then incubated with 50 µL of SSNP measurement buffer (6 M LiCl, 10 mM Tris, 1 mM EDTA; pH 8) for elution. Samples were continuously mixed on a rotary mixer during incubation and elution steps. Incubation and elution times were varied as indicated in the text.

### SSNP measurements and analyses

SSNP measurements and analyses were conducted as previously described.^23^ Briefly, SSNPs consisted of a single pore in a 20 or 30 nm thick low stress silicon nitride membrane that was fabricated using Helium ion milling^24^ or obtained commercially (Norcada, Inc.). All pores used in measurements displayed a linear current‐voltage curve with resistance values indicating a diameter in the range of 7.3–10.4 nm as calculated from an established model.^25^ An effective pore thickness of 1/3 membrane thickness was assumed.^25^ Each nanopore was rinsed with ethanol and water, dried with filtered air, and treated with air plasma (30 W, Harrick Plasma) for at least 2 min per side before being mounted into a custom three‐dimensional‐printed flow cell (Carbon, Inc.). Measurement buffer (6 M LiCl, 10 mM Tris, 1 mM EDTA, pH 8.0) was then introduced to the flow cell to contact both sides of the nanopore membrane. Ag/AgCl electrodes were used to connect to a patch‐clamp amplifier (Axopatch 200B, Molecular Devices) for electrical measurement.

10 µL of HA eluate in measurement buffer was loaded on one side of the pore. While the concentration of extracted HA was not measured explicitly, the functional range of concentrations^13^ relevant to SSNP analysis is generally 1–100 ng/µL. A 300 mV bias was applied, and the trans‐membrane current was monitored at a rate of 200 kHz using a 100 kHz four‐pole Bessel filter. Data were collected and analyzed using a custom LabVIEW program (National Instruments). An additional 5 kHz low‐pass filter was applied during analysis. Molecular translocations were marked by temporary reductions in the ionic current and were considered for analysis if beyond 6 standard deviations of the root‐mean‐square noise of the baseline.^14^ The dwell time and average conductance amplitude for each translocation event were used to calculate an event charge deficit (ECD)^13^ through which a corresponding MW was determined using an average calibration curve that was produced by measuring multiple quasi‐monodisperse samples of HA with known MWs on different SSNPs as described previously.^23^ We note that without an internal calibration, device‐to‐device differences in pore diameter relative to the average may have induced some additional variance in distribution metrics between experiments. Events corresponding to MWs between 50 kDa and 10 MDa were considered in the analysis. MW distribution histograms were generated with a bin width of 0.08 on a log_10_‐transformed kDa scale. Details of all analyses are provided in Supporting Information: Table S1.

### Microfluidic device fabrication and operation

AutoCAD software was used to generate microfluidic patterns. Polymethyl methacrylate (PMMA, 1.3 mm thickness, McMaster‐Carr) was patterned using a commercial laser etcher (Glowforge, Inc.). Adhesive films (0.15 mm thickness, 9495MPF, 3 M) were used to seal layers of PMMA. The fluidic device contained channels (0.5 mm width) composing a central chamber flanked on both sides by serpentine channels for additional on‐chip fluid volume (1 mL total capacity) terminating at edge ports into which lengths of silicon tubing were inserted and sealed with instant bonding ethyl adhesive (Loctite® 495; Henkel Adhesive Technologies) as a primary inlet and outlet for fluid delivery. A secondary inlet provided a reduced path length to the center chamber through which magnetic beads could be delivered directly. Port entries were beveled to ensure a well‐sealed system. Diametrically magnetized cylindrical neodymium magnets (D48DIA; K&J Magnetics, Inc.) were introduced above and below the central chamber to provide a magnetic field to capture functionalized beads within the device. Cutouts in the outermost PMMA layers ensured consistent placement of the magnets with a 1.6 mm vertical distance between them. Total device dimensions were 75 × 25 × 7.1 mm. A microperistaltic pump (MP,^2^ Elemental Scientific, Inc.) was used to control flow rates and was operated via a custom LabVIEW program.

The entire device was first flushed for 5 min with 1× PBS at 5 µL/s via the peristaltic pump. 50 µL of functionalized VG1‐beads at a concentration of 10 mg/mL (see Section *Affinity extraction of HA*) was then introduced to the central channel via the secondary inlet at 0.5 µL/s leaving beads visibly captured within the magnetic field. The secondary inlet was then closed and 1× PBS was introduced at 0.5 µL/s for 5 min via the primary inlet to wash the stationary beads and remove excess beads from the system. 50 µL of polydisperse HA (300 ng/µL in 1× PBS) was introduced via the primary inlet at 2 µL/s behind a small (~1 mm) gap of air to prevent mixing with the clean buffer. The sample was pushed until all wash buffer was flushed from the system through the outlet and discarded. The HA fluid volume was then cycled repetitively back and forth for 1 h through the stationary field of beads via the peristaltic pump at a flow rate of 0.5 µL/s. 1× PBS was then introduced via the primary inlet at 0.5 µL/s for 5 min behind an air gap to flush the unbound HA out of the outlet and wash the beads. Air was pushed into the primary inlet until it reached the secondary inlet, and the primary inlet was sealed. Finally, 25 µL of measurement buffer was introduced via the secondary inlet behind an air gap and cycled repetitively through the beads at 0.5 µL/s for 1 h to elute HA. The HA eluate was recovered via the outlet for subsequent SSNP analysis.

## RESULTS AND DISCUSSION

The elution of HA in the bead‐based affinity extraction technique requires interrupting the interaction between the binding element VG1 and its target. Typically, this has been achieved through irreversible means that are destructive to the VG1 and in some cases could also have impacts on captured molecules. For example, a high temperature (95°C) has been used to denature VG1 thermally, enabling efficient liberation of HA.^13^ However, refolding of the protein is not possible and there is evidence^26^ that such treatment can impact the stability or structure of the released HA, potentially in a sample‐dependent manner. Similarly, denaturing chemical eluents like sodium dodecyl sulfate or urea could be used to release captured molecules, but along with drawbacks like those listed above, removal of these additives for subsequent processes can be challenging. An ideal alternative approach would release HA efficiently with conditions that: (i) do not permanently damage the VG1, (ii) do not impact the HA, and (iii) are compatible with downstream analyses.

**Figure 1:**
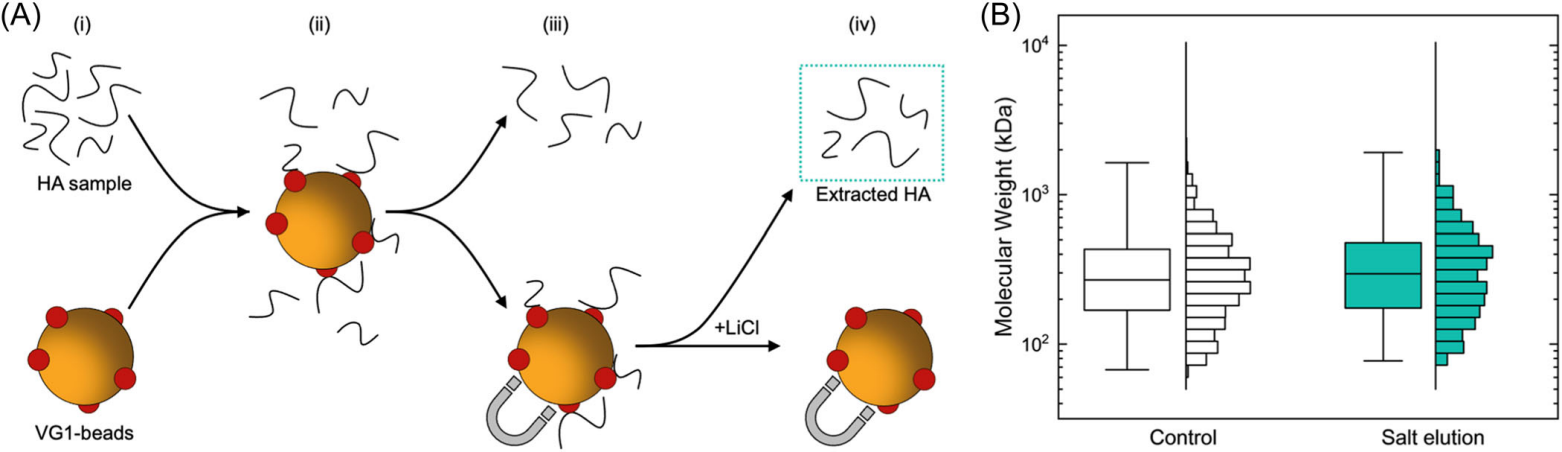
(A) Schematic of the HA extraction protocol. VG1 beads and sample of HA (i) are mixed to capture a subset of HA (ii). HA‐bound beads are magnetically separated from unbound HA (iii) and are then incubated with high‐molarity LiCl to elute captured HA (iv) for subsequent analysis. (B) SSNP MW distribution boxplot and histogram for polydisperse HA before (*Control*: *N* = 1472 events; median 270 kDa, IQR 169–432 kDa) and after extraction (*Salt elution*: *N* = 474 events; median 296 kDa, IQR 175–477 kDa). HA, hyaluronan; IQR, interquartile range; SSNP, solid‐state nanopore.

To achieve these optimization goals, we modified our extant affinity extraction strategy to culminate in elution with high salt conditions (Figure 1A). Like other protein‐ligand interactions, VG1 recognition for HA is governed by the surface charge, shape, and hydrophobicity of the binding surface,^27^ which combine to promote highly‐specific attachment to HA. Elevated salt conditions impact this recognition in several ways associated with more efficient charge screening. First, a higher counter‐ion screening alters the apparent surface charge of the binding region directly. Similarly, charge screening can reduce interactions between amino acids in the protein itself, loosening its structure and consequently impacting stericity. Finally, ionic interactions increase the surface tension around the protein via repulsion of co‐ions due to the electrostatic image force, thereby altering hydrophobic effects at the solute–solvent interface.^28^ For these reasons, VG1‐HA interactions are typically assayed at low (~100 mM) salt concentrations.^29^ However, the capacity of elevated salt concentration to disrupt the binding of HA to VG1 positions it as an efficient method of elution; particularly for SSNP analysis since high ionic strength is required for assay performance.^14^

As an initial demonstration, we first incubated a polydisperse HA sample (see Section *Materials and Methods*) with VG1‐conjugated superparamagnetic beads for 1 h and then eluted with 6 M LiCl for 1 h at room temperature. The Li^+^ cation was chosen partially because we expected its small size to facilitate invasion between VG1 and HA and disrupt the interaction to allow the release of HA into the buffer, but also because we have previously reported 6 M LiCl as an optimized condition for SSNP analysis of HA.^14^ Consequently, its utilization enabled direct assessment of eluted HA without further processing after magnetic removal of the VG1‐beads. For validation, we performed SSNP analysis of the salt‐eluted HA and generated a MW distribution that could be compared directly to that of control HA that did not undergo extraction. As shown in Figure 1B, the resulting distributions were in excellent agreement with the median of the extracted population (296 kDa) matching closely to that of the control (270 kDa). This demonstrated that the incorporation of high salt elution into the extraction process yielded the efficient release of HA with a size profile representative of the starting material.

**Figure 2:**
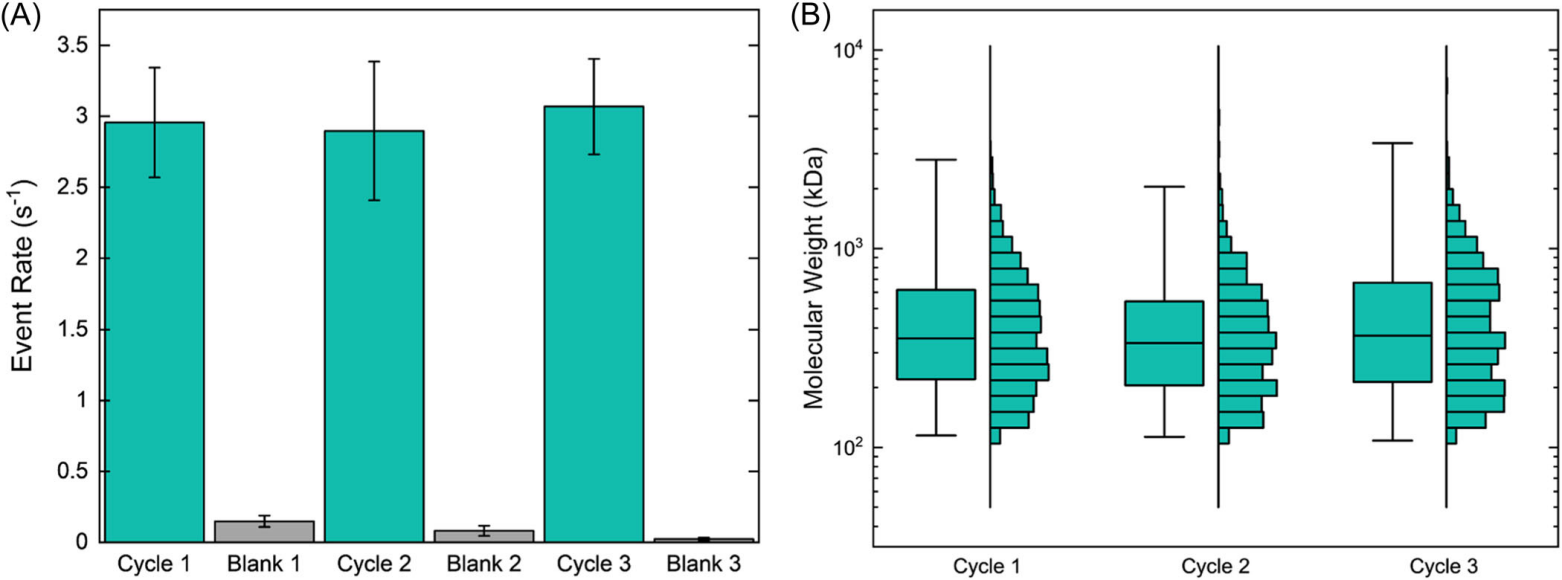
(A) SSNP event rates measured for iterative extractions of polydisperse HA including extracted HA (*Cycles 1–3*; 2.96 ± 0.39 s^−1^, 2.90 ± 0.49 s^−1^, 3.07 ± 0.34 s^−1^, respectively) and no‐HA blanks (*Blanks 1–3*; 0.15 ± 0.04 s^−1^, 0.08 ± 0.04 s^−1^, 0.02 ± 0.01 s^−1^, respectively). (B) SSNP MW distribution boxplots and histograms for extracted HA samples. *Cycle 1*: *N* = 1985 events; median 354 kDa, IQR 221–618 kDa. *Cycle 2*: *N* = 728 events; median 335 kDa, IQR 206–543 kDa. *Cycle 3*: *N* = 1285 events; median 366 kDa, IQR 214–672 kDa. HA, hyaluronan; IQR, interquartile range; SSNP, solid‐state nanopore.

Because of the transient nature of ionic effects, the changes induced in the VG1 binding pocket should be reversible. Consequently, a simple post‐elution wash of the beads with low salt buffer is predicted to restore their binding affinity and enable their use in subsequent extraction cycles. Such iterative extraction would provide a significant advantage to the overall process by reducing costs and processing times. To test this hypothesis, we extracted aliquots of polydisperse HA standard three separate times using the same set of beads across extraction cycles. Between cycles, we alternated with “blanks” for which the entire extraction protocol was performed using fresh 6 M LiCl buffer containing no HA. This process provided a check for crosstalk resulting from incomplete HA release or nonspecific interactions. Because SSNP event rate is known to correspond with analyte concentration in solution,^13^ we used this value as a metric to assess relative elution yield. We observed a consistent event rate for all three HA extractions (average 2.97 ± 0.09 s^−1^) (Figure 2A, blue). While we cannot use this value to determine eluted HA explicitly, we can estimate from past measurements^13^ that it corresponds to a concentration of about 5 ng/µL. Given the elution volume of 50 µL, this suggests a capacity of approximately 250 ng for the amount of VG1 beads used here. Comparing with the initial mass of HA provided to the beads, the resulting capture yield is approximately 25% for these measurements. In contrast to the extractions, the blanks all produced negligible event rates (average 0.08 ± 0.06 s^−1^) (Figure 2A, gray) that were indistinguishable from the background noise floor (0.14 ± 0.15 s^−1^), confirming that no detectable material was retained on the beads between extraction cycles. As a result, each extraction could be considered an independent analysis without significant cross‐contamination. We again observed strong sample to sample correspondence in the MW distributions of extracted HA (Figure 2B) with median values of the second and third iteration varying on average only approximately 4% from the first extraction, comparing favorably with the average variation between replicates with our SSNP devices (~5%). This finding demonstrated high measurement reproducibility and suggested that reused VG1‐beads were functionally indistinguishable from fresh ones in extracting HA. Critically, these iterative extractions also demonstrated that high ionic strength conditions did not impact the incredibly stable biotin‐streptavidin bond used to conjugate VG1 with the beads, as suggested by past literature that has identified a stabilizing effect of monovalent salts on the bond.^30^

**Figure 3:**
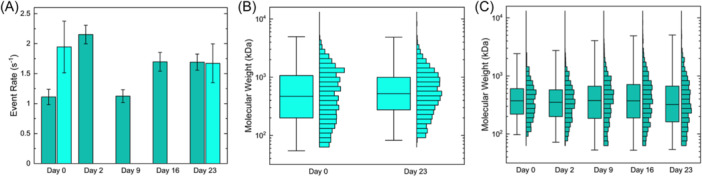
(A) Comparison of SSNP event rates measured for two sets of polydisperse HA extractions performed iteratively across 23 days: HA extracted two times (light) and five times (dark). Left to right: 1.11 ± 0.13 s^−1^, 1.94 ± 0.43 s^−1^, 2.15 ± 0.15 s^−1^, 1.12 ± 0.11 s^−1^, 1.70 ± 0.16 s^−1^, 1.69 ± 0.13 s^−1^, and 1.67 ± 0.32 s^−1^. (B) SSNP MW distribution boxplots and histograms for HA extracted twice in the timespan, on Day 0 (*N* = 1518 events; median 371 kDa, IQR 158–847 kDa) and Day 23 (*N* = 1326 events; median 413 kDa, IQR 218–788 kDa). (C) SSNP MW distribution boxplots and histograms for HA extracted five times in the timespan, on Day 0 (*N* = 474 events; median 296 kDa, IQR 175–477 kDa), Day 2 (*N* = 1370 events; median 280 kDa, IQR 158–457 kDa), Day 9 (*N* = 1079 events; median 299 kDa, IQR 147–528 kDa), Day 16 (*N* = 1413 events; median 295 kDa, IQR 150–564 kDa), and Day 23 (*N* = 1307 events; median 256 kDa, IQR 128–529 kDa. HA, hyaluronan; IQR, interquartile range; SSNP, solid‐state nanopore.

To explore the limits of this VG1‐bead reuse approach, we repeated the extraction process at regular intervals over a 3‐week period from single aliquots of VG1‐beads using polydisperse HA. To separate time from a number of cycles, one bead aliquot was used to extract HA only twice—once on Day 0 and once on Day 23—while another was used to extract five times at regular intervals over the same time span. Analyzing the SSNP event rates for each time point (Figure 3A), we observed minor variation in the net amount of HA retrieved across cycles (average 1.63 ± 0.39 s^−1^) but found no indication of VG1 deterioration; indeed, the event rate for VG1‐beads undergoing five extractions was slightly higher on the last extraction than the first extraction. The event rates observed here were somewhat less than in the iterative extractions described above, suggesting a slightly lower capture efficiency that may have been driven by differences in bead mass, VG1 conjugation, or even SSNP dimensions. As above, the MW distributions associated with these measurements showed consistency across iterations as well; for the two‐extract set, the median value of the final extraction varied by approximately 11% compared to its initial extraction (Figure 3B) while medians for the five‐extract set varied by approximately 5% on average compared to their initial extraction (Figure 3C). Collectively, these data suggest that, in addition to the salt elution process inducing no irreversible damage to the VG1, the overall extraction performance was stable across a 3‐week timescale and across a significant number of extraction cycles.

**Figure 4:**
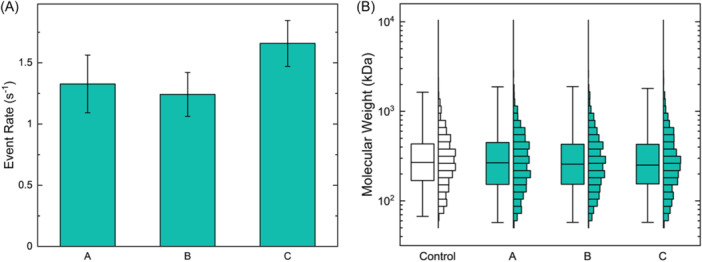
(A) SSNP event rates for HA extracted at 2–8°C with 15‐min incubation steps. Technical replicates A–C: 1.33 ± 0.24 s^−1^, 1.24 ± 0.18 s^−1^, and 1.66 ± 0.19 s^−1^. (B) SSNP MW distribution boxplots and histograms for the control polydisperse HA (*N* = 1472 events; median 270 kDa, IQR 169–432 kDa) and three replicates of the extracted HA: *A* (*N* = 1040; median 267 kDa, IQR 153–448 kDa), *B* (*N* = 1214 events; median 258 kDa, IQR 153–429 kDa), and *C* (*N* = 1184 events; median 251 kDa, IQR 155–428 kDa). HA, hyaluronan; IQR, interquartile range; SSNP, solid‐state nanopore.

With no measurable performance deterioration of the beads observed across extraction cycles, VG1 lifetime emerged as the limiting factor to consider in iterative bead reuse. Therefore, we next sought to minimize the negative impacts of the extraction process on VG1 stability. As a first consideration, the extant protocol involves multiple 1‐h incubation steps where VG1 beads may be exposed to sample matrices that could in principle contain fouling agents or protein degraders that could potentially reduce average VG1 activity and constrain the possible number of extractions from a single set of beads. Consequently, limiting incubation times could be expected to extend the overall VG1 lifetime. In addition, the stability of proteins is typically maximized under controlled conditions that include limited exposure to ambient temperatures (e.g., room temperature). While the activity of VG1 can be expected to maintain for at least 6 months when stored at −80°C according to the manufacturer, the minimum allowable temperature after conjugation is dictated by the superparamagnetic beads themselves, which cannot be frozen but should instead be stored at 2–8°C to maximize stability. Consequently, further lifetime improvements could also be expected by performing all extraction steps at low temperature. However, the consequences of any protocol changes could have performance repercussions. For example, HA making less interaction contacts or exhibiting lower potential for avidity to the VG1‐beads could be expected to elute faster, and since high MW molecules have the opportunity bind to multiple elements along their contour length, incomplete elution could result in a distribution bias. Additionally, the use of low‐temperature conditions could alter the dynamics of the capture or elution process, either of which could impact the yield of HA or induce biases. As a demonstration, we altered the protocol by limiting incubation times for both capture and elution to 15 min and reducing the temperature of all steps to 2–8°C. The full modified extraction protocol was performed in triplicate to evaluate reproducibility. The resulting eluates provided sufficient material to produce robust event rates (average 1.41 ± 0.22 s^−1^; Figure 4A) that indicate a similar yield to that stated above and the resulting MW distributions were in excellent agreement with the control distribution ( ~ 4% average variability of medians compared to control; Figure 4B). These measurements demonstrated that both the incubation and elution time used during the affinity extraction protocol could be reduced by 75% and could be carried out under refrigeration without a notable impact on performance.

While our investigations establish a pathway for long‐term, iterative use of VG1‐beads in HA extraction, one potential challenge for the implementation of the immunoprecipitation‐like approach is the mechanism of capture. In free solution, HA capture by VG1‐beads occurs via a diffusive mechanism because binding can happen only when the two molecular partners are close enough to interact. Given that the VG1 is anchored to relatively large beads (2.8 µm diameter), this process would be driven largely by the HA with its much higher diffusivity. However, the varying amounts of HA found in diverse biological matrices^4^ necessitates extraction from solutions having different HA concentrations, and the dynamics of interaction in low‐concentration media would diverge from that in high‐concentration media. These effects could ultimately result in inconsistent minimum capture timescales or even size biases induced by MW‐dependent differences in HA diffusion coefficients.^31^ One possible solution would be the employment of a factor other than diffusion to drive molecular capture. Guided by this principle and with potential process automation in mind, we finally sought to implement a microfluidic approach wherein HA delivery to the VG1 was controlled via flow conditions rather than passive diffusion.

**Figure 5:**
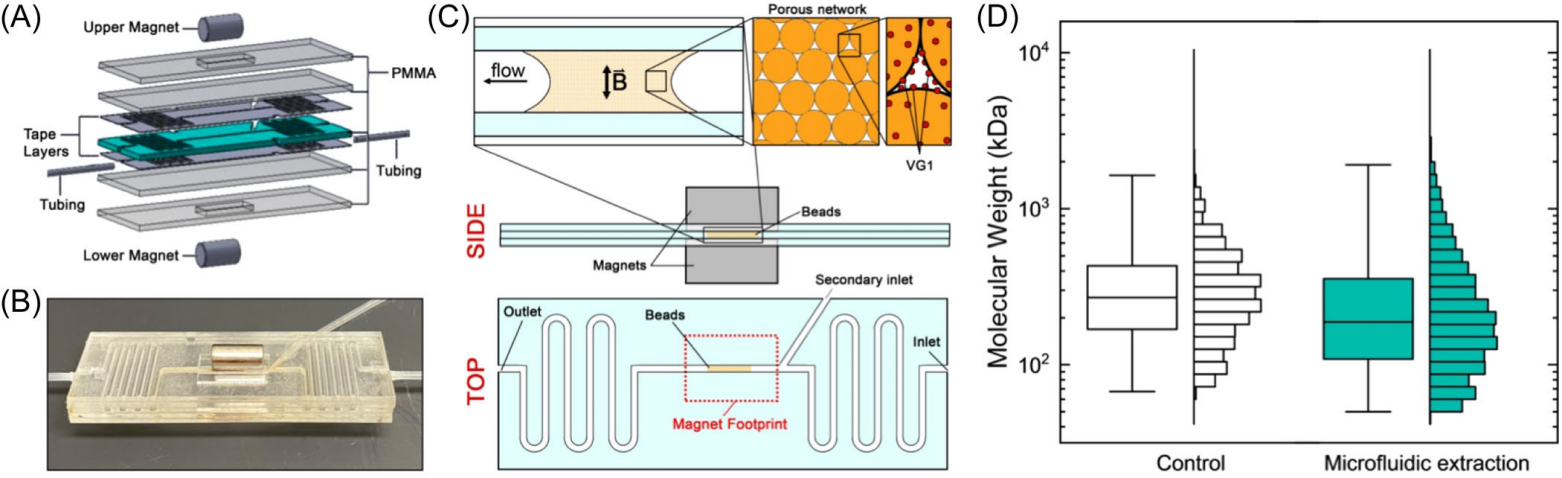
(A) Exploded view of the microfluidic HA device layers. (B) Photograph of the assembled microfluidic device with tubing connected and magnets positioned above and below the center chamber. (C) Schematic representation of the assembled microfluidic device. Side view (above) shows magnetic trap surrounding a channel. Insets (top) show zooms of bead field trapped in the magnetic field (left), the porous network they form (center), and the exposed VG1 elements for capturing HA (right). Top view (below) shows main channel for analyte capture/release as well as inlets/outlet and magnet footprint. (D) SSNP MW distribution boxplot and histogram for control polydisperse HA (*N* = 1472 events; median 270 kDa, IQR 169–432 kDa) and polydisperse HA extracted with the microfluidic device (*N* = 1245 events; median 188 kDa, IQR 109–355 kDa). HA, hyaluronan; IQR, interquartile range; SSNP, solid‐state nanopore.

Our platform (Figure 5A) consisted of stacked layers of PMMA, and adhesive polymeric films patterned to produce a single chamber with two inlets, one outlet, and serpentine channels designed to expand on‐chip fluid capacity. At the center of the structure, permanent magnets were positioned above and below the channel with their poles in diametric opposition (Figure 5B). Device dimensions enabled the magnets surrounding the flow channel to be separated by only 1.6 mm, creating a strong magnetic field inside the center chamber. Injection of VG1‐beads into the inlet with the shorter path length with a direct feed into the center of the device (secondary inlet) resulted in their immobilization at the center of the magnet position (Figure 5C). Following prior work,^32^ the two‐magnet configuration ensured a distribution of the beads across the entire channel width between the magnets and created a static array through which sample volumes could then be flowed. We note that under visual inspection, nearly all beads introduced to the device were retained within the magnetic field with no significant population of uncaptured beads and no significant amount of release due to flow. We did observe a small amount of bead loss due to nonspecific interactions with the inside of the inlet tubing during injection, but we estimate that this could account for no more than 5% of the total bead mass.

Molecular capture was performed by introducing HA solution into the serpentine region on one side of the device and flowing the mixture through the magnetically arrested VG1 beads. Because the beads were in a static‐packed array, their binding sites could sample only from a limited interstitial volume. This enabled fluid flow rather than passive diffusion to be the dominant factor guiding the delivery of additional HA material to the VG1, rather than being limited by diffusion. To test an extreme of concentration, we used 300 ng/µL polydisperse HA and flowed it through the system at a rate of 0.5 µL/s. When nearly the full sample fluid volume had cycled past the VG1 beads, flow direction was reversed. This reciprocating behavior was repeated for 1 h to facilitate HA binding before eluting with 6 M LiCl for 1 h in situ and retrieving the resulting solution. HA captured with this flow‐limited microfluidic architecture yielded a high SSNP event rate (6.55 ± 0.14 s^−1^). When considered along with the reduced elution volume used here (25 μL), this rate value indicates a similar bead capacity to the free‐solution capture described above but represents a significantly lower capture yield ( ~ 2%) due to the high initial HA concentration. The MW distribution was in general agreement with the original distribution (Figure 5D) but with a shift in the extracted distribution (median 188 kDa) relative to the control (median 270 kDa). This disparity could be the result of a capture bias resulting from the extreme concentration probed here or of shearing‐induced fragmentation of bound HA caused by relative motion of the captured beads. In addition, it could be partially due to device‐to‐device differences in SSNP dimensions^14^ since only an average calibration value was employed for all data here. Moreover, the observed difference suggests that additional tuning of experimental variables like maximum HA concentration, flow rate, capture time, elution time, and bead packing density may be considered for further process optimization. Regardless, our results demonstrate an alternative approach to bead‐based HA extraction that is semi‐automated and may be amenable to high‐throughput applications. By employing flow as the HA delivery vehicle, the device should also be able to accommodate low‐concentration specimens that would be challenged by long diffusion kinetics in solution‐based capture. Additionally, the magnetically arrested VG1 beads can be washed and reused without removing them from the device, and the platform itself is amenable to operation under cold conditions to extend VG1 lifetime as above.

In conclusion, we have described several critical improvements to the affinity extraction of HA. We first demonstrated that HA can be eluted efficiently from VG1 beads using high salt conditions that do not denature the protein and enable iterative capture and elution from the same VG1 beads without significant crosstalk. We then implemented several approaches to extending the lifetime of the protein component of the VG1 beads by reducing both capture and elution time and verifying that the process can be performed in the low‐temperature (2–8°C) conditions preferred for protein storage. Together, these innovations yield processes that increase efficiency and lower costs while maintaining high fidelity for HA size distribution analyses. Finally, we developed a proof‐of‐concept semi‐automated HA extraction process using a microfluidic device designed to replace diffusion with fluid flow as the rate‐limiting capture step in delivering HA sample to VG1 binding domains. While we challenged the device with a very high HA concentration (300 ng/µL) here and observed some size bias, lower concentrations could be employed easily as could extreme dilutions of high‐concentration mixtures, provided that the flow rate enables sampling over a reasonable time. Ongoing studies with this device will focus on optimizing conditions to maximize capture fidelity to position the flow‐regulated delivery of HA to VG1 capture elements as a rapid and high‐capacity extraction approach. Overall, our results establish a pathway to improve HA extraction performance that can also be applied easily to other target biomolecules. In the context of extraction from complex biological matrices, additional optimizations may be required, for example, an extremely low HA concentration or the presence of an abundance of competing biomolecules may alter the kinetics of capture and result in a longer minimum incubation time than in our synthetic specimens. However, the same principles can be applied to those protocols to optimize translational performance. In future work, greater precision in SSNP analysis of extracted material can be achieved through the implementation of internal calibration standards to minimize the role of pore‐to‐pore variability.

## AUTHOR CONTRIBUTIONS

Dorothea A. Erxleben contributed to experimental design, performed measurements, analyzed the data, and wrote the manuscript. Felipe Rivas designed and implemented the microfluidic device, performed extractions and measurements, and contributed to overall experimental design. Ian Smith performed extractions and measurements for bead optimization studies and contributed to experimental design. Suruchi Poddar performed measurements and contributed to experimental design. Paul L. DeAngelis synthesized quasi‐monodisperse HA material. Elaheh Rahbar contributed to experimental design. Adam R. Hall oversaw the project, contributed to experimental design, and wrote the manuscript. All authors contributed to the editing and review of the manuscript.

## CONFLICT OF INTEREST STATEMENT

A.R.H, P.L.D, and E.R are listed as inventors on a patent covering SSNP analysis of HA.

## ETHICS STATEMENT

No human‐ or animal‐derived specimens were used in this work.

## Supporting information

Supplementary information.

## Data Availability

The data that support the findings of this study are available from the corresponding author upon reasonable request.
