# Supplementary material for: High‐fidelity and iterative affinity extraction of hyaluronan
Source: Proteoglycan Res. 2024 Dec 6;2(4):e70008. doi: 10.1002/pgr2.70008 (PMC11623434; doi:10.1002/pgr2.70008)
Supplement: Supplementary file 1 — Supplementary information. [file PGR2-2-e70008-s001.pdf]

Supplementary Information: High-fidelity and iterative affinity extraction of hyaluronan

Dorothea A. Erxleben, Felipe Rivas, Ian Smith, Suruchi Poddar, Paul L. DeAngelis, Elaheh Rahbar, Adam R. Hall

Data location	# events	Median (kDa)	Q1 (kDa)	Q3 (kDa)	Mean (kDa)	Stdev of Mean	Event Rate (s^{-1})	Stdev of Rate
Figure 1b: Control<br>Figure 4b: Control<br>Figure 5d: Control	1472	270	169	432	337	243	-	-
Figure 1b: Salt elution<br>Figure 3c: Day 0	474	296	175	477	375	285	1.11	0.13
Figure 2: Cycle 1	1985	354	221	618	479	380	2.96	0.39
Figure 2: Cycle 2	728	335	206	543	433	351	2.90	0.49
Figure 2: Cycle 3	1285	366	214	672	508	434	3.07	0.34
Figure 2a: Blank 1	-	-	-	-	-	-	0.15	0.04
Figure 2a: Blank 2	-	-	-	-	-	-	0.08	0.04
Figure 2a: Blank 3	-	-	-	-	-	-	0.02	0.01
Figure 3b: Day 0	1518	371	158	847	583	573	1.94	0.43
Figure 3b: Day 23	1326	413	218	788	581	532	1.67	0.32
Figure 3c: Day 2	1370	280	158	457	356	279	2.15	0.15
Figure 3c: Day 9	1079	299	147	528	405	386	1.12	0.11
Figure 3c: Day 16	1413	295	150	564	442	483	1.70	0.16
Figure 3c: Day 23	1307	256	128	529	402	429	1.69	0.13
Figure 4: A	1040	267	153	448	344	275	1.33	0.24
Figure 4: B	1214	258	153	429	339	273	1.24	0.18
Figure 4: C	1184	251	155	428	335	279	1.66	0.19
Figure 5d: Microfluidic extraction	1245	188	109	355	301	323	6.55	0.14

Table S1. Descriptive metrics for all SSNP data presented in the manuscript.
